# Risk Stratification for Early Neurological Deterioration After Mechanical Thrombectomy and Development of a 24-h Postprocedural Reassessment Model in Patients With Acute Anterior-Circulation Large-Vessel Occlusion Based on Computed Tomography Perfusion Mismatch Volume

**DOI:** 10.31083/RN53745

**Published:** 2026-08-26

**Authors:** Wei Du, Aiping He, Linxu Jiang

**Affiliations:** ^1^Department of Neurosurgery, The Hongda Hospital of Jiamusi University, 154000 Jiamusi, Heilongjiang, China; ^2^Department of Neurosurgery, Huai’an Clinical Medical College of Jiangsu University, Huai’an Hospital of Huai’an City, 223200 Huai’an, Jiangsu, China; ^3^Department of Emergency, The Fifth Affiliated Hospital of Xinjiang Medical University, 830011 Urumqi, Xinjiang, China

**Keywords:** ischemic stroke, thrombectomy, perfusion imaging, clinical deterioration, risk assessment

## Abstract

**Background:**

Following mechanical thrombectomy for acute ischemic stroke due to anterior-circulation large-vessel occlusion, early neurological deterioration (END) continues to be a clinically consequential complication. Computed tomography perfusion (CTP)-derived mismatch volume estimates the amount of hypoperfused tissue that is not part of the ischemic core, but its value for END stratification at different time points has not been clarified. We therefore examined the relationship between mismatch volume and END and constructed risk models anchored to clearly specified temporal windows.

**Methods:**

Consecutive patients treated with mechanical thrombectomy between January 2021 and December 2024 were retrospectively included. END encompassed either a ≥4-point increase from the preprocedural National Institutes of Health Stroke Scale (NIHSS) score or death from any cause during the first 72 h after thrombectomy; deaths occurring within 72 h were analyzed as END events. Potential nonlinearity was examined using restricted cubic spline (RCS) analysis. Only information known before thrombectomy was entered into the preprocedural baseline model. For the 24-h landmark analysis, patients who remained free of END through 24 h formed the reassessment cohort; successful recanalization status and hemorrhagic transformation subtypes were added to predict delayed END arising after >24–72 h. Discrimination, calibration, clinical utility, and internal validity were evaluated by receiver operating characteristic (ROC) analysis, calibration assessment, decision curve analysis (DCA), and 1000 bootstrap resamples, respectively.

**Results:**

END occurred in 87 of 438 patients (19.9%): 78 experienced neurological worsening and 9 died within 72 h. The association between mismatch volume and END departed from linearity (*p* for nonlinearity = 0.023). Observed END frequencies were 33.0%, 18.9%, and 12.5% for mismatch volumes <75 mL, 75–105 mL, and >105 mL, respectively (*p* for trend <0.001). Mismatch volume, ischemic core volume, admission NIHSS score, and collateral score constituted the preprocedural model, which produced an area under the ROC curve (AUC) of 0.789 (95% confidence interval [CI], 0.735–0.843) and a bootstrap-corrected AUC of 0.774. END occurred in 8.0%, 20.7%, and 38.5% of patients assigned to the low-, intermediate-, and high-risk categories of the simplified preprocedural score. Among 387 patients eligible for 24-h reassessment, 36 developed delayed END; the corresponding model yielded an AUC of 0.838 (95% CI, 0.773–0.903). Delayed END was associated with parenchymal hematoma but not with hemorrhagic infarction.

**Conclusions:**

CTP mismatch volume was related to END following mechanical thrombectomy. The preprocedural and 24-h reassessment models, each defined by its prediction time, retained acceptable discrimination after internal validation; independent external validation remains necessary.

## 1. Introduction

Among patients with acute ischemic stroke, occlusion of a major anterior-circulation artery frequently results in severe disability or death. Neurological status may worsen soon after treatment, and such early neurological deterioration (END) adversely influences both early and later outcomes [1]. No universal definition has been adopted for either the observation period or the magnitude of change on the National Institutes of Health Stroke Scale (NIHSS); nevertheless, a rise of at least 4 points or death during the 72 h following thrombectomy is widely used to identify clinically relevant deterioration [2]. Computed tomography perfusion (CTP) supplies quantitative estimates of the ischemic core, overall hypoperfusion, and potentially salvageable tissue, making it informative for acute imaging evaluation [3]. After mechanical thrombectomy, target-mismatch patterns and the numerical difference between hypoperfusion and core volumes have been linked to tissue fate and functional recovery, whereas their usefulness for predicting END is not yet established [4]. The prognostic meaning of perfusion mismatch may vary with ischemic core size, suggesting that these two measures should be considered together [5]. Collateral blood flow influences how rapidly ischemic injury progresses and how long penumbral tissue persists; it therefore contributes to outcome variation even when the occlusion site is similar [6]. Accordingly, assessment of collateral status by clinical and imaging methods is an established element of acute-stroke risk evaluation [7]. Hemorrhagic transformation after reperfusion is another potential contributor to END, but its impact may differ according to subtype and time of onset [8]. In addition, several published prediction models combine information obtained before and after treatment without clearly specifying when prediction is made, thereby risking temporal bias [9]. The present study assessed the association of CTP mismatch volume with END after mechanical thrombectomy and developed two temporally separated tools: a baseline model restricted to preprocedural variables and a reassessment model applied at 24 h. These models were intended as an exploratory framework for subsequent testing in external cohorts [10].

## 2. Materials and Methods

### 2.1 Study Population

**Fig. 1.**
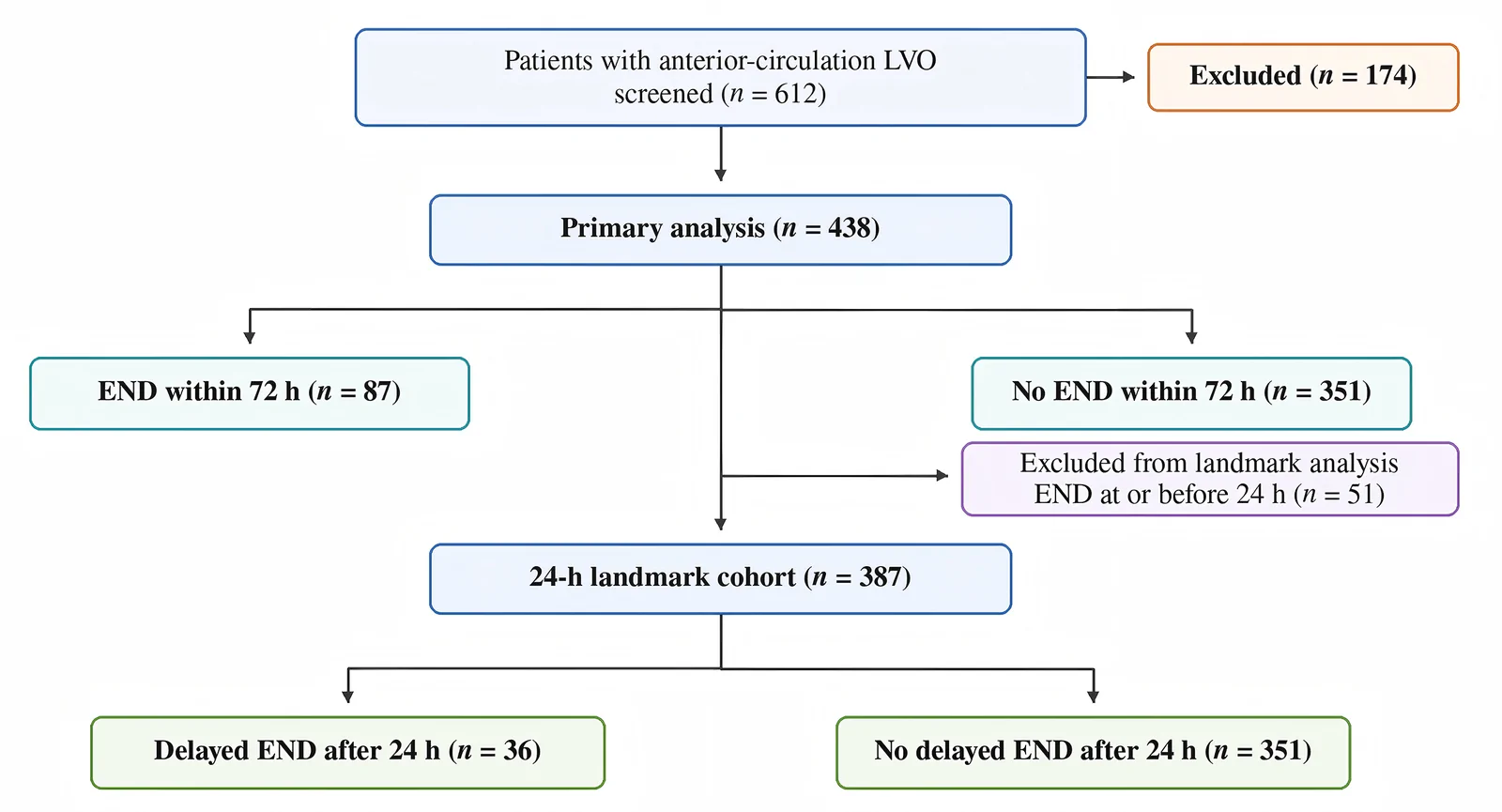
**Patient selection flowchart**. Of 612 consecutively screened patients, 174 were excluded: posterior-circulation occlusion (*n* = 68), no preprocedural computed tomography perfusion (CTP) or unanalyzable images (*n* = 39), prestroke modified Rankin Scale (mRS) score >2 (*n* = 22), severe comorbidity or terminal malignancy (*n* = 15), concomitant intracranial hemorrhage/tumor or another severe intracranial lesion (*n* = 10), onset-to-puncture time >24 h (*n* = 7), and inability to ascertain both the 72-h National Institutes of Health Stroke Scale (NIHSS) score and survival status (*n* = 13). The primary analysis included 438 patients; all 9 deaths within 72 h were retained and classified as early neurological deterioration (END). The 24-h landmark cohort excluded 51 patients who had developed END at or before 24 h and included 387 patients, of whom 36 developed delayed END after 24 h and through 72 h. LVO, large-vessel occlusion.

At a single center, we retrospectively assembled a consecutive cohort of patients treated with mechanical thrombectomy for acute ischemic stroke caused by anterior-circulation large-vessel occlusion between January 2021 and December 2024. Eligibility required all of the following: (1) age ≥18 years; (2) intracranial internal carotid artery (ICA) or M1/M2 middle cerebral artery (MCA) occlusion verified by computed tomography angiography (CTA) or magnetic resonance angiography (MRA); (3) no more than 24 h from symptom onset to femoral-artery puncture; (4) preprocedural CTP of sufficient quality for postprocessing; (5) completion of mechanical thrombectomy; and (6) available NIHSS assessments and survival information through 72 h after the intervention. Patients were excluded for (1) posterior-circulation occlusion; (2) absent preprocedural CTP or images unsuitable for analysis; (3) coexisting intracranial hemorrhage, tumor, or another serious intracranial disorder; (4) a prestroke modified Rankin Scale (mRS) score >2; (5) severe cardiac, hepatic, or renal dysfunction or terminal malignancy; (6) an onset-to-puncture interval >24 h; or (7) inability to determine both the 72-h NIHSS score and survival status because of transfer, discharge against medical advice, or another reason. Death during the first 72 h did not result in exclusion; these patients remained in the cohort and were assigned to the END group. Among 612 screened patients, 174 met an exclusion criterion: posterior-circulation occlusion (*n* = 68), unavailable or unanalyzable preprocedural CTP (*n* = 39), prestroke mRS score >2 (*n* = 22), severe comorbidity or terminal malignancy (*n* = 15), concomitant intracranial hemorrhage/tumor or another severe intracranial lesion (*n* = 10), onset-to-puncture time >24 h (*n* = 7), or unavailable 72-h NIHSS and survival data (*n* = 13). The final cohort therefore comprised 438 patients (Fig. 1). This study was reported in accordance with the STROBE statement (**Supplementary STROBE Checklist**).

### 2.2 Computed Tomography Perfusion Protocol

**Fig. 2.**
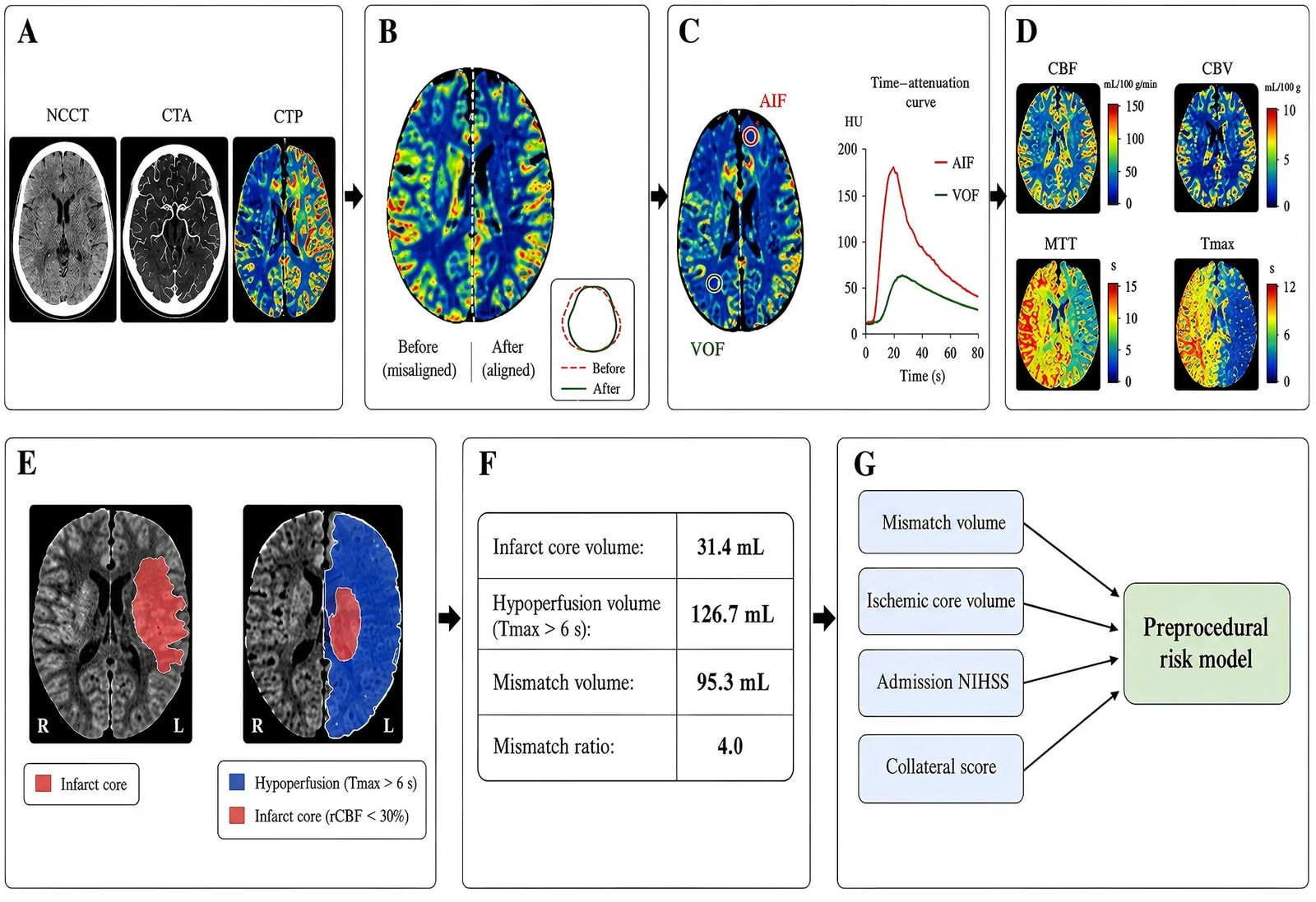
**Schematic workflow for CTP image postprocessing**. (A–G) show image input, motion correction, arterial input function (AIF)/venous output function (VOF) selection, perfusion maps, threshold-based segmentation, quantitative output, and variables entered into the risk model, respectively. The illustrative values are an ischemic core volume of 31.4 mL, a time-to-maximum (Tmax) >6-s hypoperfusion volume of 126.7 mL, a mismatch volume of 95.3 mL, and a mismatch ratio of 4.0. NCCT, noncontrast computed tomography; CTA, computed tomography angiography; CBF, cerebral blood flow; CBV, cerebral blood volume; MTT, mean transit time; rCBF, relative cerebral blood flow; R, right; L, left. In panel (E), the red area represents the ischemic core (rCBF <30%), and the blue area represents the hypoperfused region (Tmax >6 s).

Before thrombectomy, each patient underwent noncontrast computed tomography (NCCT), CTP, and CTA. Perfusion acquisition used 80 kV, 150 mAs, 5-mm sections, and cranial coverage of 80–100 mm. Iohexol (Omnipaque 350, 350 mg I/mL, SKU Y-542, GE Healthcare (Shanghai) Co., Ltd., Shanghai, China) was injected at 0.5–0.7 mL/kg at 4–6 mL/s. Data collection started 4 s after injection and lasted 50–60 s. RAPID version 5.1.1 (iSchemaView, Menlo Park, CA, USA) automatically processed the CTP series and produced maps of cerebral blood flow (CBF), cerebral blood volume (CBV), mean transit time (MTT), and time-to-maximum (Tmax) [11]. Tissue with relative cerebral blood flow (rCBF) <30% was designated as ischemic core, whereas a Tmax delay >6 s defined the hypoperfused region. Mismatch volume equaled hypoperfusion volume minus ischemic core volume; mismatch ratio equaled hypoperfusion volume divided by ischemic core volume. The Alberta Stroke Program Early CT Score (ASPECTS) was assigned using the established scoring method [12]. CTA collateral status was rated with the Tan scale, on which scores 0–1 denoted poor and scores 2–3 denoted good collaterals [13]. As illustrated in Fig. 2, the CTP workflow proceeded from NCCT, CTA, and dynamic CTP input through motion correction, arterial input function (AIF) and venous output function (VOF) selection, perfusion-map generation, threshold-based segmentation, and calculation of mismatch metrics.

### 2.3 Mechanical Thrombectomy and Periprocedural Management

All thrombectomy procedures were undertaken by credentialed neurointerventionalists with relevant experience. Transfemoral access was the usual approach, and the operator selected stent retrieval, direct aspiration, or their combination according to lesion characteristics. Reperfusion was scored on the modified Thrombolysis in Cerebral Infarction (mTICI) scale; grades 2b–3 were considered successful [14]. Airway protection, agitation, and hemodynamic condition were jointly reviewed by the neurointerventional and anesthesia teams when choosing the anesthetic strategy [15]. Following the procedure, patients received at least 24 h of observation in a neurological intensive care unit (ICU) or stroke unit. Blood pressure goals were tailored to reperfusion status, bleeding risk, and the overall clinical state. In general, after successful recanalization the center aimed for systolic blood pressure (SBP) of 120–140 mmHg and sought to avoid sustained values ≥160 mmHg; when reperfusion was unsuccessful or reocclusion was a concern, SBP was generally maintained at 140–180 mmHg [16,17]. Antithrombotic treatment was ordinarily withheld during the first 24 h in patients who had undergone intravenous thrombolysis. Follow-up NCCT was routinely obtained at approximately 24 h, and CTA was added when clinically warranted. Once intracranial hemorrhage had been ruled out, initiation of antiplatelet or anticoagulant therapy was individualized according to stroke mechanism, stent placement, and bleeding risk.

### 2.4 Definition of Early Neurological Deterioration

An END event was recorded if the NIHSS score increased by ≥4 points from its preprocedural baseline at any assessment within 72 h after mechanical thrombectomy or if the patient died from any cause within 72 h after the procedure. NIHSS scoring was performed by neurologists who had completed standardized training and certification. Planned evaluations occurred before thrombectomy and 2, 24, 48, and 72 h afterward; additional examinations were performed whenever clinically required. For patients who died within 72 h, mortality determined END status and no postmortem NIHSS value was imputed. A patient who fulfilled the ≥4-point worsening criterion before death contributed only one END event.

### 2.5 Data Collection

Data abstracted from electronic medical records included demographics, vascular risk factors, admission clinical findings, laboratory measurements, imaging characteristics, and treatment-related information. On imaging, hemorrhagic transformation (HT) was categorized as hemorrhagic infarction (HI) or parenchymal hematoma (PH), with subcategories HI1, HI2, PH1, and PH2; separating HI from PH is supported by their differing clinical relevance [18]. Two physicians independently reviewed postprocedural hemorrhagic findings, and a third senior physician resolved discordant classifications. The use of a time-specific assessment after thrombectomy is further supported by evidence from dual-energy computed tomography (CT) and 24-h follow-up imaging [19].

### 2.6 Statistical Analysis

IBM SPSS Statistics version 26.0 (IBM Corp., Armonk, NY, USA) and R version 4.2.0 (R Foundation for Statistical Computing, Vienna, Austria) were used for all analyses. Depending on distribution, continuous data are reported as mean ± standard deviation or median (interquartile range); groups were compared with an independent-samples *t* test or the Mann–Whitney *U* test. Categorical data are given as number (percentage) and were tested with the *χ^2^* test or Fisher exact test. Key variables were complete, and missingness for every other covariate was below 10%. Little’s test produced *p* = 0.418, so the hypothesis that data were missing completely at random (MCAR) was not rejected. For the primary analysis, missing values were handled under a missing-at-random assumption by multiple imputation by chained equations (MICE; *m* = 20). The imputation model contained the outcome, all candidate predictors, and variables related to missingness, and Rubin’s rules were used to combine estimates. **Supplementary Table 1** describes missing-data patterns; **Supplementary Table 2** presents complete-case and worst-case sensitivity analyses. A restricted cubic spline (RCS) was fitted to examine whether the mismatch volume–END relationship was nonlinear. Predictor eligibility was determined by the time at which information became available. Thus, the baseline model was limited to preprocedural variables, whereas the 24-h landmark model omitted patients who had experienced END by 24 h and treated END developing >24–72 h as the outcome. Candidate covariates were specified in advance from prior evidence, clinical importance, and availability at the relevant prediction time. Model 1 estimated each candidate predictor without adjustment. Model 2 included age, sex, diabetes mellitus, atrial fibrillation, and random blood glucose at admission. Model 3 further incorporated mismatch-volume category, ischemic core volume, admission NIHSS score, and collateral score. Because ASPECTS and ischemic core volume were collinear, ischemic core volume was retained owing to its central role in the study hypothesis. Collinearity was considered present when the variance inflation factor (VIF) exceeded 5. Model discrimination was quantified from receiver operating characteristic (ROC) curves and the area under the ROC curve (AUC). Calibration was examined with calibration plots and the Hosmer–Lemeshow test, while decision curve analysis (DCA) was used to explore net clinical benefit. Internal validation relied on 1000 bootstrap resamples. As a sensitivity analysis, least absolute shrinkage and selection operator (LASSO) logistic regression was fitted with 10-fold cross-validation, and results were reported for both the penalty minimizing cross-validation error (*λmin*) and the more parsimonious 1-standard-error penalty (*λ1se*). Statistical tests were two-sided, and *p* < 0.05 defined statistical significance. Results from analyses without multiplicity adjustment were regarded as exploratory.

## 3. Results

### 3.1 Baseline Characteristics

Within the cohort of 438 patients, 87 met the END definition and 351 remained free of END. These events consisted of 78 patients with NIHSS deterioration of ≥4 points who survived through 72 h and 9 patients who died within 72 h. Relative to patients without END, those with END were older, more frequently had diabetes mellitus or atrial fibrillation, and presented with higher admission NIHSS scores and random blood glucose concentrations (all *p* < 0.05). Sex, body mass index (BMI), hypertension, prior stroke/transient ischemic attack (TIA), coronary artery disease, smoking, alcohol use, admission SBP, admission diastolic blood pressure (DBP), and onset-to-admission interval showed no statistically significant group differences (all *p* > 0.05) (Table 1).

**Table 1.** **Comparison of Demographic characteristics, vascular risk factors, and clinical data at admission between the two groups**.

Variable	END group (*n* = 87)	Non-END group (*n* = 351)	Test statistic	*p* value
Age, y, mean ± SD	71.3 ± 10.8	67.5 ± 11.4	*t* = 2.81	0.005
Male, *n* (%)	49 (56.3)	198 (56.4)	*χ^2^* < 0.01	0.988
BMI, kg/m^2^, mean ± SD	24.1 ± 3.2	23.8 ± 3.4	*t* = 0.77	0.441
Hypertension, *n* (%)	58 (66.7)	217 (61.8)	*χ^2^* = 0.70	0.403
Diabetes mellitus, *n* (%)	35 (40.2)	98 (27.9)	*χ^2^* = 5.00	0.025
Atrial fibrillation, *n* (%)	41 (47.1)	124 (35.3)	*χ^2^* = 4.13	0.042
Previous stroke/TIA, *n* (%)	15 (17.2)	52 (14.8)	*χ^2^* = 0.32	0.574
Coronary artery disease, *n* (%)	18 (20.7)	58 (16.5)	*χ^2^* = 0.84	0.358
Smoking history, *n* (%)	31 (35.6)	139 (39.6)	*χ^2^* = 0.46	0.496
Alcohol use, *n* (%)	22 (25.3)	101 (28.8)	*χ^2^* = 0.42	0.517
SBP, mmHg, mean ± SD	152.6 ± 23.5	149.8 ± 22.1	*t* = 1.05	0.294
DBP, mmHg, mean ± SD	85.3 ± 14.2	84.1 ± 13.8	*t* = 0.72	0.470
Admission NIHSS score, median (IQR)	18 (14–22)	14 (10–18)	*Z* = 5.12	<0.001
Random blood glucose at admission, mmol/L, mean ± SD	8.9 ± 3.4	7.8 ± 2.9	*t* = 2.67	0.008
Onset-to-admission time, min, mean ± SD	186.5 ± 98.7	178.3 ± 102.4	*t* = 0.68	0.497

Note: BMI, body mass index; TIA, transient ischemic attack; SBP, systolic blood pressure; DBP, diastolic blood pressure; SD, standard deviation; IQR, interquartile range. Data are presented as mean ± SD, median (IQR), or number (percentage) [*n* (%)]. *t* denotes the independent-samples *t*-test statistic; *Z*, the standardized Mann–Whitney *U*-test statistic; *χ^2^*, the Pearson chi-square statistic; and Fisher, Fisher exact test. All *p* values are two-sided; *p* < 0.05 was considered statistically significant.

### 3.2 Laboratory Variables

Compared with the non-END group, the END group had higher white blood cell (WBC) counts, fasting glucose, glycated hemoglobin A1c (HbA1c), and D-dimer values (all *p* < 0.05). Hemoglobin, platelet count, total cholesterol (TC), triglycerides (TG), low-density lipoprotein cholesterol (LDL-C), high-density lipoprotein cholesterol (HDL-C), creatinine, international normalized ratio (INR), and fibrinogen were similar between groups (all *p* > 0.05) (Table 2).

**Table 2.** **Comparison of laboratory variables between the two groups**.

Variable	END group (*n* = 87)	Non-END group (*n* = 351)	Test statistic	*p* value
WBC count, ×10^9^/L, mean ± SD	9.8 ± 3.6	8.9 ± 3.2	*t* = 2.13	0.035
Hemoglobin, g/L, mean ± SD	132.5 ± 18.3	135.2 ± 17.6	*t* = 1.24	0.217
Platelet count, ×10^9^/L, mean ± SD	198.6 ± 62.4	205.3 ± 58.7	*t* = 0.91	0.366
Fasting blood glucose, mmol/L, mean ± SD	7.6 ± 2.8	6.7 ± 2.3	*t* = 3.12	0.002
HbA1c, %, mean ± SD	6.8 ± 1.5	6.4 ± 1.3	*t* = 2.49	0.013
TC, mmol/L, mean ± SD	4.52 ± 1.08	4.38 ± 1.02	*t* = 1.09	0.276
TG, mmol/L, median (IQR)	1.42 (0.98–1.95)	1.38 (0.95–1.88)	*Z* = 0.68	0.496
LDL-C, mmol/L, mean ± SD	2.78 ± 0.86	2.69 ± 0.82	*t* = 0.88	0.380
HDL-C, mmol/L, mean ± SD	1.12 ± 0.32	1.15 ± 0.29	*t* = 0.80	0.427
Creatinine, μmol/L, mean ± SD	82.5 ± 24.6	79.8 ± 22.3	*t* = 0.99	0.321
INR, mean ± SD	1.05 ± 0.18	1.03 ± 0.15	*t* = 1.08	0.281
Fibrinogen, g/L, mean ± SD	3.52 ± 0.98	3.38 ± 0.92	*t* = 1.25	0.211
D-dimer, mg/L, median (IQR)	1.86 (0.92–3.54)	1.28 (0.68–2.45)	*Z* = 2.54	0.011

Note: WBC, white blood cell; HbA1c, glycated hemoglobin A1c; TC, total cholesterol; TG, triglycerides; LDL-C, low-density lipoprotein cholesterol; HDL-C, high-density lipoprotein cholesterol; INR, international normalized ratio. Data are presented as mean ± SD or median (IQR). *t* denotes the independent-samples *t*-test statistic; *Z*, the standardized Mann–Whitney *U*-test statistic; *χ^2^*, the Pearson chi-square statistic; and Fisher, Fisher exact test. All *p* values are two-sided.

### 3.3 Imaging Variables

Patients with END showed a greater ischemic core volume but smaller mismatch volume, a lower mismatch ratio, poorer collateral scores, and lower ASPECTS than patients without END (all *p* < 0.001). Neither hypoperfusion volume nor occlusion location differed significantly (both *p* > 0.05). The proportion developing END declined stepwise as mismatch-volume category increased (*p* for trend <0.001) (Table 3).

**Table 3.** **Comparison of imaging variables between the two groups**.

Variable	END group (*n* = 87)	Non-END group (*n* = 351)	Test statistic	*p* value
Occlusion site, *n* (%)	ICA 28 (32.2); M1 46 (52.9); M2 13 (14.9)	ICA 89 (25.4); M1 201 (57.3); M2 61 (17.4)	*χ^2^* = 1.70	0.427
Ischemic core volume, mL, median (IQR)	43.2 (26.5–67.8)	22.4 (11.5–39.2)	*Z* = 6.32	<0.001
Hypoperfusion volume, mL, median (IQR)	118.5 (88.2–165.4)	112.6 (79.8–150.2)	*Z* = 0.86	0.390
Mismatch volume, mL, median (IQR)	71.8 (47.5–101.6)	88.5 (61.8–117.6)	*Z* = 3.98	<0.001
Mismatch ratio, median (IQR)	2.8 (1.9–4.0)	4.9 (3.1–7.0)	*Z* = 5.62	<0.001
Collateral score, *n* (%)	Score 0: 12 (13.8); score 1: 28 (32.2); score 2: 32 (36.8); score 3: 15 (17.2)	Score 0: 18 (5.1); score 1: 62 (17.7); score 2: 152 (43.3); score 3: 119 (33.9)	*χ^2^* = 21.83	<0.001
ASPECTS, median (IQR)	6 (5–8)	8 (7–9)	*Z* = 5.42	<0.001
Mismatch-volume stratum, *n* (%)	<75 mL: 35 (40.2); 75–105 mL: 31 (35.6); >105 mL: 21 (24.1)	<75 mL: 71 (20.2); 75–105 mL: 133 (37.9); >105 mL: 147 (41.9)	Overall *χ^2^* = 17.34; trend *χ^2^* = 16.40	<0.001

Note: ICA, internal carotid artery; ASPECTS, Alberta Stroke Program Early CT Score. Data are presented as median (IQR) or number (percentage) [*n* (%)]. *Z* denotes the standardized Mann–Whitney *U*-test statistic; *χ^2^*, the Pearson chi-square statistic; overall *χ^2^*, the overall comparison among strata; and trend *χ^2^*, the Cochran–Armitage linear trend test statistic. All *p* values are two-sided.

### 3.4 Periprocedural and Treatment-Related Variables

General anesthesia, more thrombectomy passes, unsuccessful recanalization, any HT, and PH within 24 h were more frequent in the END group (all *p* < 0.05). No significant difference was observed for receipt of intravenous thrombolysis (IVT), type or dose of IVT agent, onset-to-IVT interval, onset-to-puncture interval, puncture-to-reperfusion interval, intra-arterial medication, HI, antithrombotic initiation within 24 h, or duration of monitoring (all *p* > 0.05). During the first 24 h after thrombectomy, patients with END had higher mean and peak SBP, more episodes exceeding 180 mmHg, and less time within the designated blood pressure range (all *p* < 0.05) (Table 4).

**Table 4.** **Comparison of periprocedural and treatment-related variables between the two groups**.

Periprocedural/treatment variable	END group (*n* = 87)	Non-END group (*n* = 351)	Test statistic	*p* value
Anesthetic strategy, *n* (%)	General anesthesia 52 (59.8); conscious sedation 35 (40.2)	General anesthesia 168 (47.9); conscious sedation 183 (52.1)	*χ^2^* = 3.95	0.047
Intravenous thrombolysis, *n* (%)	38 (43.7)	172 (49.0)	*χ^2^* = 0.79	0.373
IVT agent, *n* (%)	Alteplase 28 (73.7); tenecteplase 8 (21.1); urokinase 2 (5.3)	Alteplase 122 (70.9); tenecteplase 42 (24.4); urokinase 8 (4.7)	Fisher–Freeman–Halton	0.898
IVT dose, mg/kg, mean ± SD	0.86 ± 0.12	0.88 ± 0.10	*t* = 1.07	0.284
Onset-to-IVT time, min, mean ± SD	168.5 ± 52.3	162.8 ± 48.6	*t* = 0.65	0.516
Onset-to-puncture time, min, mean ± SD	285.6 ± 142.8	268.4 ± 138.5	*t* = 1.04	0.300
Puncture-to-reperfusion time, min, mean ± SD	78.5 ± 42.6	72.3 ± 38.4	*t* = 1.33	0.184
Number of thrombectomy passes, median (IQR)	3 (2–4)	2 (1–3)	*Z* = 2.65	0.008
Intra-arterial medication administration, *n* (%)	18 (20.7)	56 (16.0)	*χ^2^* = 1.11	0.291
Successful recanalization, *n* (%)	61 (70.1)	303 (86.3)	*χ^2^* = 13.05	<0.001
No HT, *n* (%)	55 (63.2)	293 (83.5)	*χ^2^* = 17.52	<0.001
HI, *n* (%)	14 (16.1)	46 (13.1)	*χ^2^* = 0.53	0.468
PH, *n* (%)	18 (20.7)	12 (3.4)	*χ^2^* = 32.59	<0.001
Mean SBP during the first 24 h after the procedure, mmHg, mean ± SD	143.6 ± 18.2	136.8 ± 15.7	*t* = 3.52	<0.001
Maximum SBP during the first 24 h after the procedure, mmHg, mean ± SD	178.5 ± 24.6	164.3 ± 22.1	*t* = 5.21	<0.001
SBP >180 mmHg, *n* (%)	18 (20.7)	35 (10.0)	*χ^2^* = 7.53	0.006
Time within target blood pressure range, %, mean ± SD	62.4 ± 21.5	71.8 ± 19.3	*t* = 3.99	<0.001
Antithrombotic therapy initiated within 24 h, *n* (%)	12 (13.8)	41 (11.7)	*χ^2^* = 0.29	0.589
Monitoring for ≥72 h, *n* (%)	82 (94.3)	335 (95.4)	Fisher	0.584

Note: IVT, intravenous thrombolysis; HT, hemorrhagic transformation; HI, hemorrhagic infarction; PH, parenchymal hematoma. Data are presented as mean ± SD, median (IQR), or number (percentage) [*n* (%)]. Percentages for IVT agent are calculated among patients who received IVT. *t* denotes the independent-samples *t*-test statistic; *Z*, the standardized Mann–Whitney *U*-test statistic; *χ^2^*, the Pearson chi-square statistic; Fisher, Fisher exact test; and Fisher–Freeman–Halton, the exact test for a 2 × k contingency table. All *p* values are two-sided.

### 3.5 Nonlinear and Correlation Analyses of Mismatch Volume

**Fig. 3.**
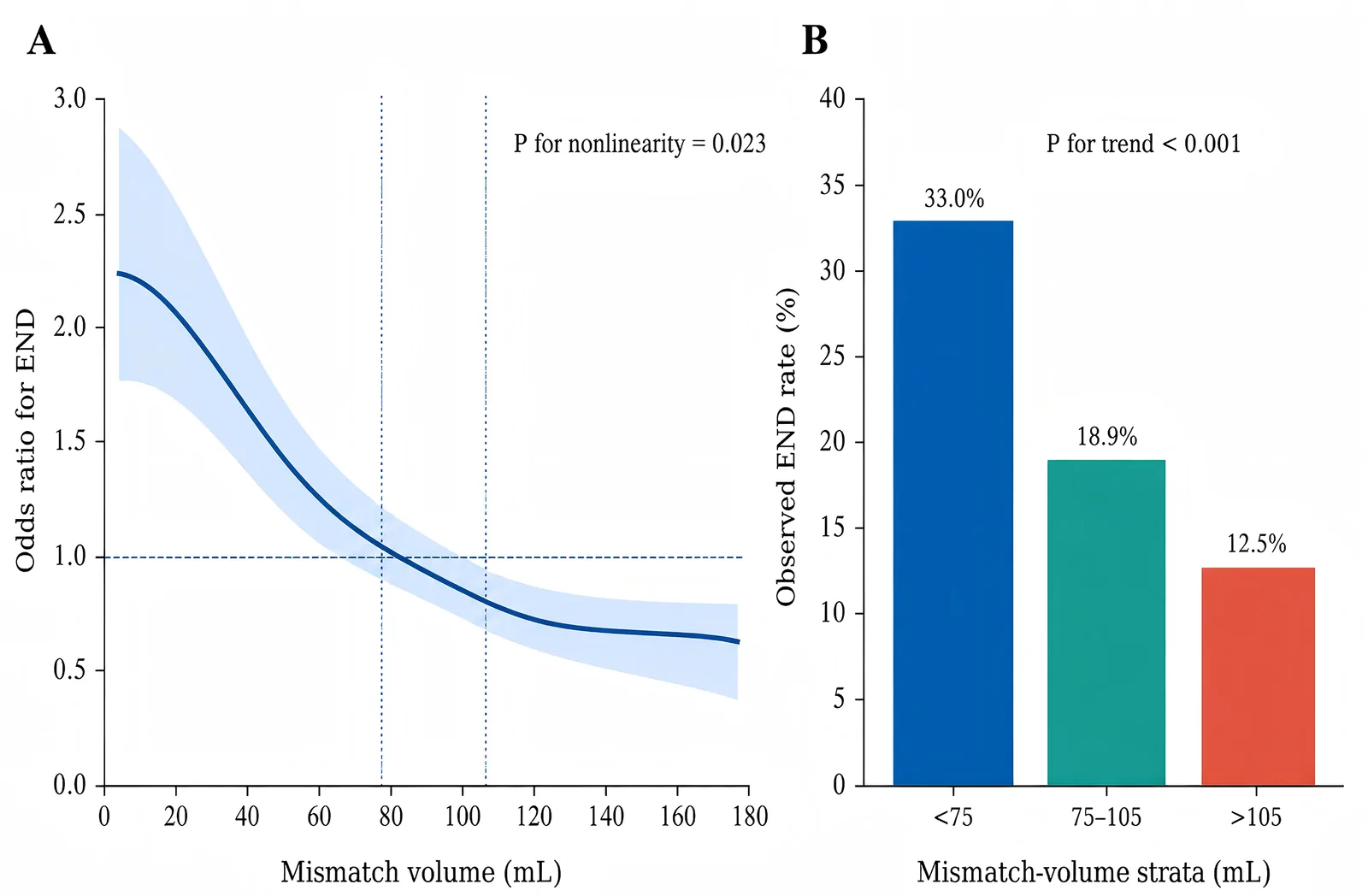
**Nonlinear association between mismatch volume and END risk and exploratory stratification**. (A) shows the restricted cubic spline (RCS) curve with 95% confidence intervals (CIs). The horizontal dashed line indicates OR = 1, and the two vertical dashed lines indicate the exploratory cut points of 75 and 105 mL; (B) shows the observed END rates in the <75-mL, 75–105-mL, and >105-mL groups.

**Fig. 4.**
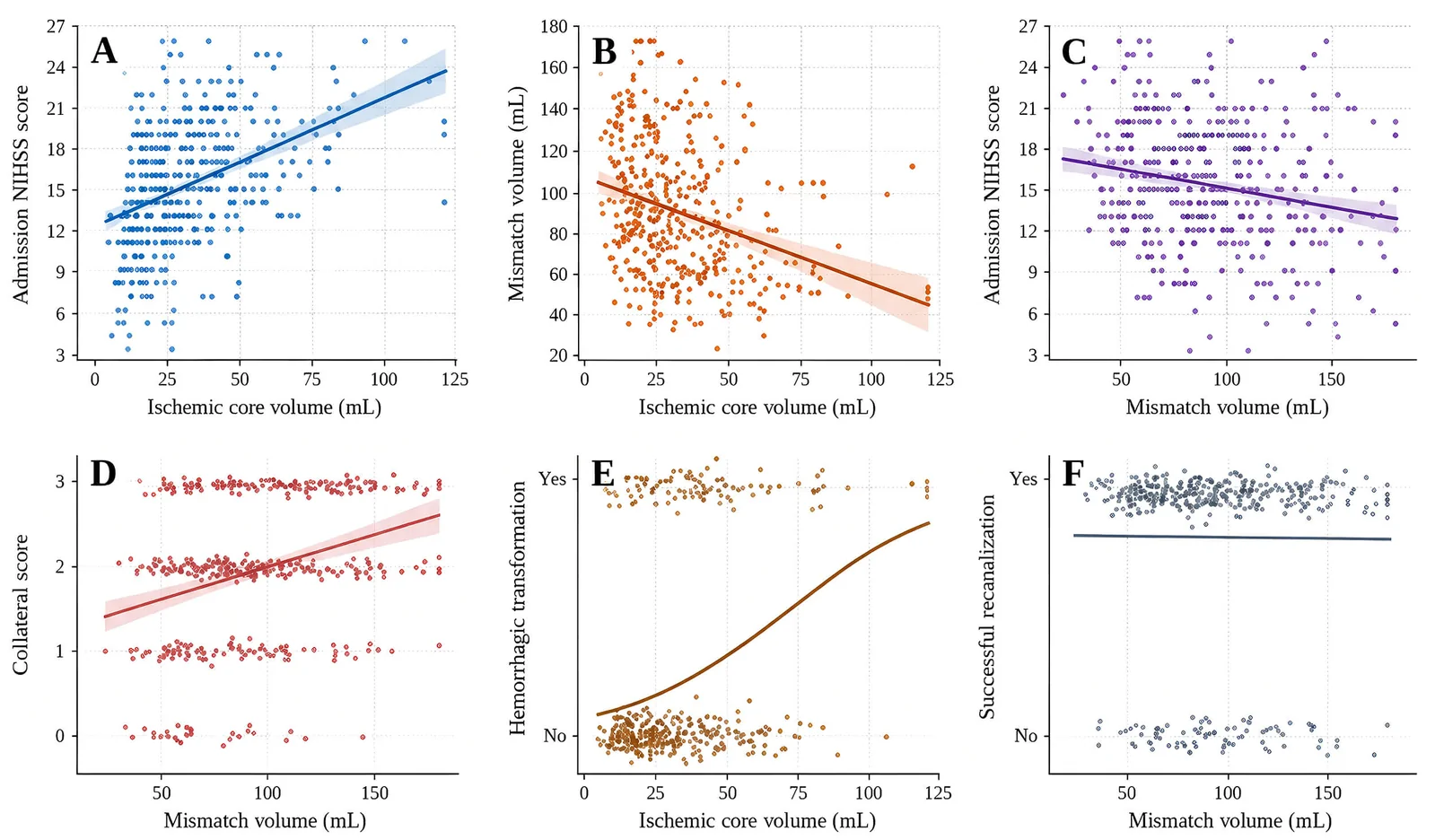
**Scatterplots and regression analyses of CTP metrics and clinical and treatment-related variables**. (A) ischemic core volume versus admission NIHSS score; (B) ischemic core volume versus mismatch volume; (C) mismatch volume versus admission NIHSS score; (D) mismatch volume versus collateral score; (E) ischemic core volume versus HT; (F) mismatch volume versus successful recanalization. (A–D) show scatterplots with fitted linear regression lines, and (E,F) show scatterplots for binary outcomes with fitted logistic regression curves; shaded areas represent 95% CIs. *ρ*, Spearman rank correlation coefficient; OR, odds ratio. A: *ρ* = 0.46, *p* < 0.001; B: *ρ* = −0.35, *p* < 0.001; C: *ρ* = −0.21, *p* < 0.001; D: *ρ* = 0.29, *p* < 0.001; E: OR per 10 mL = 1.21, *p* < 0.001; F: OR per 10 mL = 1.03, *p* = 0.217.

The RCS curve indicated that mismatch volume was related to END in a nonlinear manner (*p* for nonlinearity = 0.023). Guided by the curve shape and the risk pattern observed in this dataset, mismatch volume was divided exploratorily into <75 mL, 75–105 mL, and >105 mL. These categories summarize within-cohort risk and should not be interpreted as thresholds for treatment decisions (Fig. 3; Table 3). Correlation and regression analyses further showed a positive relationship between ischemic core volume and admission NIHSS score (Spearman *ρ* = 0.46, *p* < 0.001), an inverse relationship between ischemic core and mismatch volumes (Spearman *ρ* = −0.35, *p* < 0.001), a weak inverse relationship between mismatch volume and admission NIHSS score (Spearman *ρ* = −0.21, *p* < 0.001), and a positive relationship between mismatch volume and collateral score (Spearman *ρ* = 0.29, *p* < 0.001). Each 10-mL increase in ischemic core volume was associated with greater odds of HT (odds ratio [OR] = 1.21, *p* < 0.001). By contrast, mismatch volume was not significantly related to successful recanalization (OR per 10-mL increase = 1.03, *p* = 0.217) (Fig. 4).

### 3.6 Preprocedural Baseline Model, 24-h Reassessment Model, and Sensitivity Analyses

**Fig. 5.**
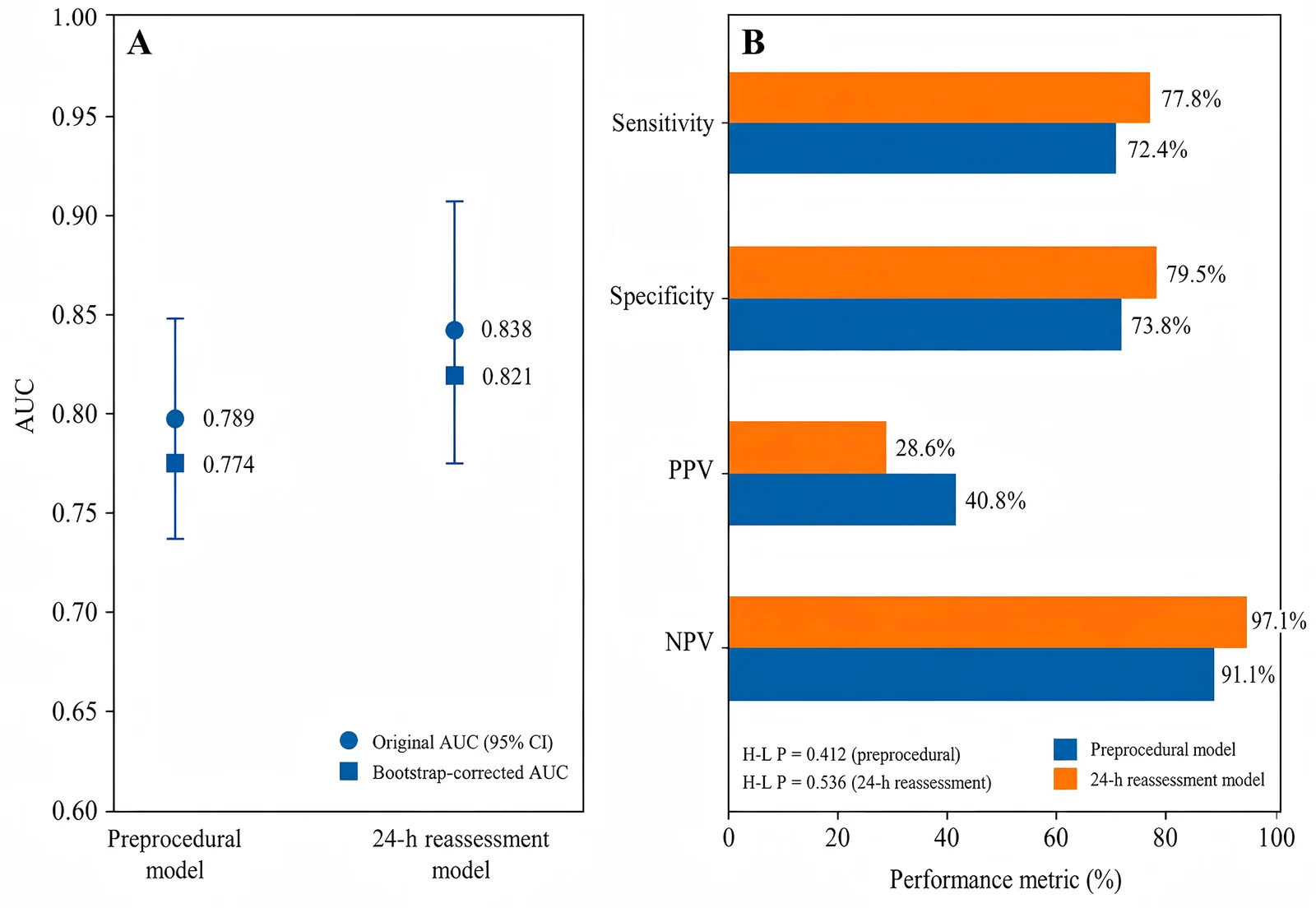
**Summary of internal performance for the preprocedural baseline and 24-h landmark reassessment models**. (A) shows the apparent area under the receiver operating characteristic curve (AUC) with 95% CIs and the bootstrap-corrected AUC. (B) shows sensitivity, specificity, positive predictive value (PPV), negative predictive value (NPV), and Hosmer–Lemeshow (H–L) test results.

**Fig. 6.**
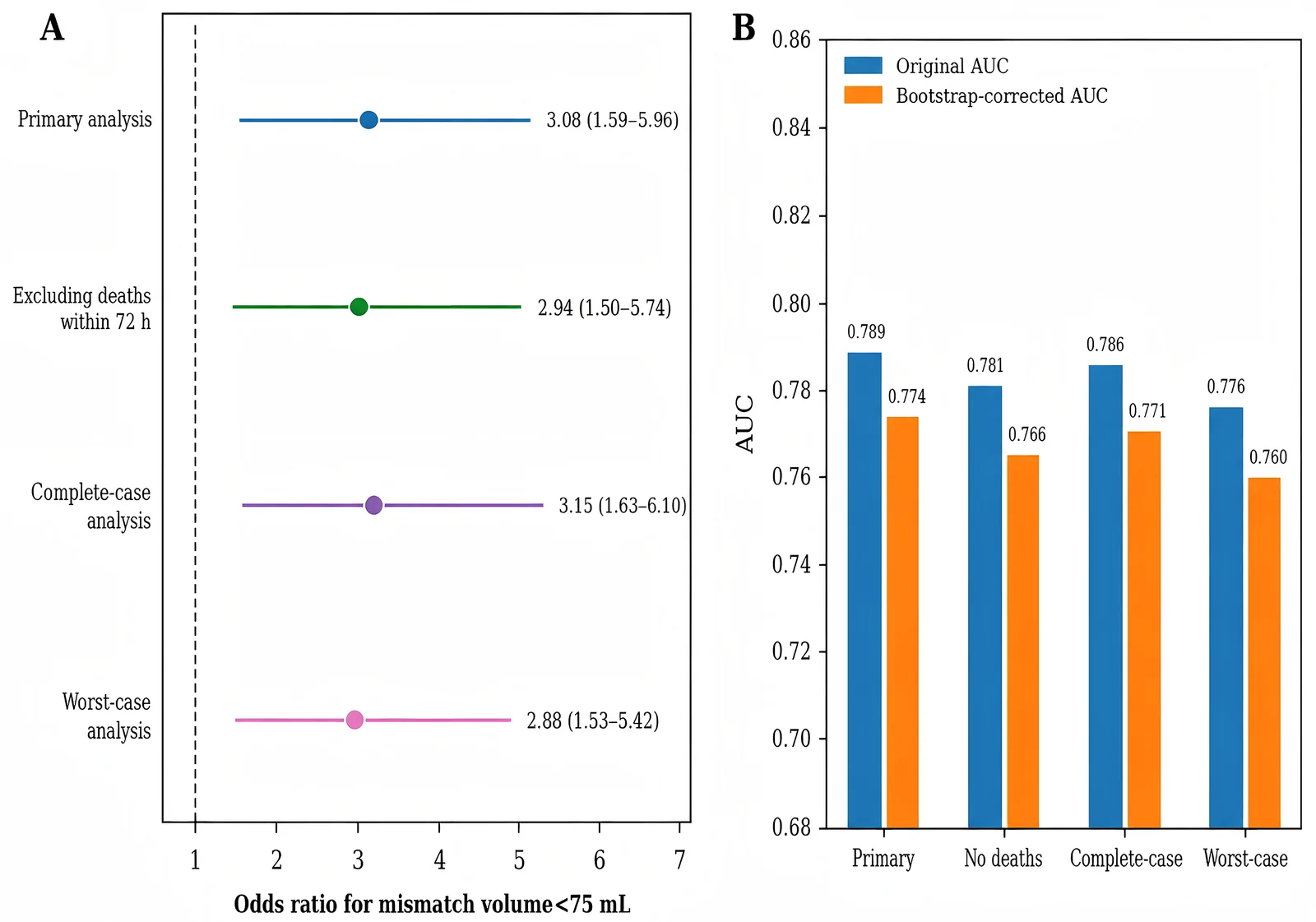
**Sensitivity analyses**. (A) shows ORs with 95% CIs for the association between mismatch volume <75 mL and END across sensitivity analyses. (B) shows the apparent and bootstrap-corrected AUCs.

**Fig. 7.**
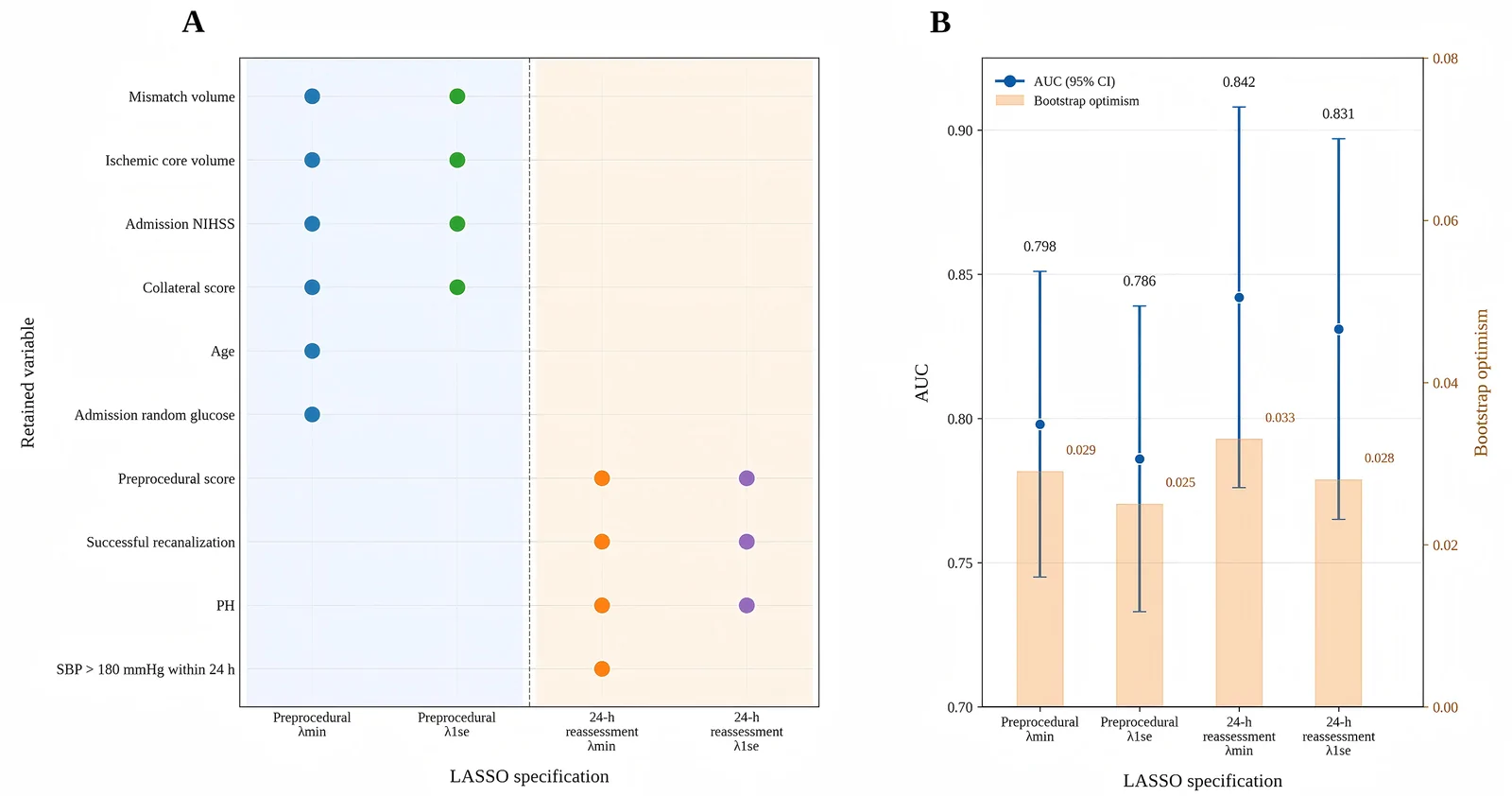
**Summary of least absolute shrinkage and selection operator (LASSO) sensitivity analyses**. (A) shows the variables retained at *λmin* and *λ1se*; (B) shows the corresponding AUCs with 95% CIs and bootstrap optimism. *λmin*, penalty parameter associated with the minimum cross-validation error; *λ1se*, more parsimonious penalty parameter selected using the 1-standard-error rule.

For mismatch volume <75 mL, ischemic core volume, admission NIHSS score, and collateral score 0–1, the direction of the estimated association was unchanged across the crude, selected-covariate-adjusted, and fully adjusted preprocedural models. In contrast, the 75–105 mL mismatch category did not retain statistical significance after full adjustment (Table 5). The apparent and bootstrap-corrected AUCs were 0.789 and 0.774 for the preprocedural model and 0.838 and 0.821 for the 24-h landmark model, respectively. In the landmark cohort, PH was related to END occurring >24–72 h, whereas HI was not (**Supplementary Table 3**; Fig. 5). Similar directions of effect were obtained when early deaths were counted as END, when deaths within 72 h were excluded, in complete-case analysis, and under the worst-case classification of indeterminate outcomes as END (Fig. 6; **Supplementary Table 2**). The LASSO analyses also retained variable patterns compatible with those of the principal preprocedural and 24-h reassessment models (Fig. 7; **Supplementary Table 4**).

**Table 5.** **Three preprocedural logistic regression models**.

Variable	Model 1: crude OR (95% CI), *p*	Model 2: selected-covariate-adjusted OR (95% CI), *p*	Model 3: fully adjusted preprocedural OR (95% CI), *p*
Mismatch volume <75 mL	3.45 (1.87–6.36), *p* < 0.001	3.21 (1.76–5.86), *p* < 0.001	3.08 (1.59–5.96), *p* = 0.001
Mismatch volume 75–105 mL	1.63 (0.89–2.98), *p* = 0.111	1.72 (0.94–3.14), *p* = 0.078	1.61 (0.88–2.96), *p* = 0.124
Ischemic core volume, per 10 mL	1.32 (1.21–1.44), *p* < 0.001	1.27 (1.15–1.41), *p* < 0.001	1.23 (1.10–1.38), *p* < 0.001
Admission NIHSS score, per 1 point	1.13 (1.08–1.18), *p* < 0.001	1.11 (1.05–1.17), *p* < 0.001	1.10 (1.04–1.16), *p* = 0.001
Collateral score 0–1	2.88 (1.73–4.80), *p* < 0.001	2.32 (1.34–4.03), *p* = 0.003	2.21 (1.25–3.91), *p* = 0.006

Note: OR, odds ratio; CI, confidence interval. Model 1 is the unadjusted model for each candidate predictor. Model 2 adjusts for age, sex, diabetes mellitus, atrial fibrillation, and random blood glucose at admission. Model 3 additionally includes the mismatch-volume strata, ischemic core volume, admission NIHSS score, and collateral score shown in the table. All three models use only variables available before thrombectomy; the ORs shown are the effect estimates for the corresponding variables in each model. All tests are two-sided; *p* < 0.05 was considered statistically significant.

### 3.7 Simplified Risk Scores

The preprocedural scoring system assigned 0–10 points from mismatch volume, ischemic core volume, admission NIHSS score, and collateral score. When patients were grouped as low risk (0–3), intermediate risk (4–6), or high risk (7–10), observed END frequency rose across successive categories. Among patients who had not experienced END by 24 h, the 24-h reassessment score added points for unsuccessful recanalization, HI, and PH, producing a possible range of 0–16. Delayed END during >24–72 h became more common from the low-risk (0–4 points) through intermediate-risk (5–8 points) to high-risk (≥9 points) categories (**Supplementary Table 4**). Because both scoring systems were created and evaluated in this cohort, they should be regarded as exploratory and not as treatment-decision cutoffs.

## 4. Discussion

With deaths during the first 72 h included in the endpoint, END occurred after mechanical thrombectomy in 19.9% of the cohort, a proportion comparable to that reported in earlier thrombectomy series [20]. Three findings warrant emphasis: the relationship between mismatch volume and END was nonlinear; the baseline and 24-h models were separated according to the time at which predictors were available; and PH was related to delayed END. The frequency of END was greatest at lower mismatch volumes. A small mismatch may signify limited salvageable tissue in the setting of a larger ischemic core and less persistent collateral support, any of which could contribute to early neurological worsening. Nevertheless, the 75- and 105-mL boundaries were derived only to describe the pattern in this cohort and cannot be interpreted as therapeutic thresholds. Associations between mismatch profiles and outcomes have also been documented after thrombectomy in prior work [21]. Ischemic core volume remained related to END in the preprocedural analysis, consistent with the broader literature linking larger cores to poorer long-term function, greater imaging-defined injury, and malignant cerebral infarction [22,23,24]. Similarly, poor collateral status may indicate reduced microvascular reserve, faster infarct expansion, and lower tolerance of ischemia. Perfusion-CT studies that quantify collateral timing and tissue outcome further support evaluating perfusion and collateral information together [25]. Postprocedural SBP and the frequency of SBP >180 mmHg were also higher among patients with END. Previous individual-patient-data meta-analysis associated blood pressure variability after thrombectomy with adverse outcomes, while randomized evidence and reviews emphasize that pressure targets must be considered alongside reperfusion status, safety, and the patient’s condition [26,27]. Accordingly, the blood pressure observations in this cohort should be viewed as associations rather than evidence that a particular target causes or prevents END.

Laboratory differences between groups involved WBC count, glucose, HbA1c, and D-dimer. Hyperglycemia may promote thromboinflammation, microvascular dysfunction, and disruption of the blood–brain barrier, potentially contributing to early worsening; however, in the present analysis these measures served mainly descriptive and confounding-control purposes and cannot establish a biological mechanism [28]. Baseline NIHSS severity, glycemic measures, and imaging burden have likewise appeared in previous END models [29]. Hemorrhagic transformation should not be treated as a single uniform entity. At the 24-h landmark, PH—but not HI—was associated with subsequent END, a pattern compatible with the greater potential of PH to exert mass effect and provoke secondary neurological decline [30]. The modest number of delayed events limits precision, so the apparent difference between HI and PH needs confirmation in larger datasets. Work based on conventional clinical predictors and machine-learning methods similarly indicates that dependable END prediction requires an explicit prediction time, variables defined before modeling, and evaluation in an external population [31]. Although conversion of a continuous model into a point score may facilitate bedside interpretation, selecting cutoffs and assessing the score in the same sample can overstate performance [32]. Future studies should therefore prioritize multicenter validation, transparent modeling, web-based implementation, and evaluation of blood biomarkers [33,34,35]. Meta-analytic findings are also consistent with END arising from a combination of demographic, clinical, laboratory, and imaging factors [36].

### Limitations

Several constraints should be considered. First, a retrospective study from one center is vulnerable to selection and information bias, residual confounding, and measurement error. Second, the mismatch-volume categories and point scores were generated within the same dataset; bootstrap validation addresses optimism only internally and cannot replace independent external assessment. Third, the 24-h landmark model is relevant only to patients who remained free of END through 24 h, and treatment or management variables may be influenced by confounding by indication. Fourth, the handling of missing values assumes data were missing at random; a nonsignificant Little’s MCAR test does not demonstrate that this assumption is true. Fifth, several association and hemorrhage-subtype analyses contained few outcome events, which increases uncertainty around the estimates. Finally, differences among CTP software platforms and acquisition or processing settings may limit transportability of the proposed mismatch-volume categories.

## 5. Conclusions

CTP mismatch volume was associated with END after mechanical thrombectomy. The preprocedural baseline model and 24-h landmark reassessment model showed acceptable discrimination after internal validation in this cohort; the thresholds, scores, and their potential applications require validation in independent multicenter cohorts.

## Data Availability

The datasets generated and analyzed during the current study are available from the corresponding author upon reasonable request.
