# Supplementary material for: Risk Stratification for Early Neurological Deterioration After Mechanical Thrombectomy and Development of a 24-h Postprocedural Reassessment Model in Patients With Acute Anterior-Circulation Large-Vessel Occlusion Based on Computed Tomography Perfusion Mismatch Volume
Source: Rev Neurol. 2026 Aug 26;81(8):53745. doi: 10.31083/RN53745 (PMC13541423; doi:10.31083/RN53745)
Supplement: Supplementary file 1 [file 1576-6578-81-8-53745-s1.zip › Supplementary Material/STROBE Statement 0825-revised.docx]

**STROBE Statement**

| **Section** | **Item No.** | **Recommendation** | **Verified page No(s)** | **Location / manuscript evidence** |
| --- | --- | --- | --- | --- |
| **Title and abstract** | 1(a) | Indicate the study's design with a commonly used term in the title or the abstract. | pp. 1-2 | The Methods portion of the abstract states that consecutive patients were retrospectively included; Section 2.1 identifies the study as a single-center retrospective cohort. |
| **Title and abstract** | 1(b) | Provide in the abstract an informative and balanced summary of what was done and what was found. | p. 1 | The abstract summarizes the background, methods, results, and conclusion, including sample size, END definition, predictors, model performance, and main conclusion. |
| **Introduction** | 2 | Explain the scientific background and rationale for the investigation being reported. | pp. 1-2 | The Introduction describes END after mechanical thrombectomy, the role of CT perfusion mismatch volume, and the need for multidimensional prediction models. |
| **Introduction** | 3 | State specific objectives, including any prespecified hypotheses. | p. 2 | The final paragraph of the Introduction states the objectives: to assess the association between CTP mismatch volume and END and to develop two time-specific tools-a preprocedural baseline model and a 24-h reassessment model. |
| **Methods** | 4 | Present key elements of study design early in the paper. | p. 2 | Section 2.1 states that this was a single-center retrospective cohort study. |
| **Methods** | 5 | Describe the setting, locations, and relevant dates, including periods of recruitment, exposure, follow-up, and data collection. | pp. 2 and 4 | Section 2.1 reports the stroke center setting and enrollment from January 2021 to December 2024; Section 2.4 reports the 72-hour postoperative outcome assessment window. |
| **Methods** | 6(a) | Cohort study - Give the eligibility criteria, and the sources and methods of selection of participants. Describe methods of follow-up. | pp. 2-4 | Section 2.1 reports inclusion/exclusion criteria and consecutive enrollment; Section 2.4 reports NIHSS assessment time points through 72 hours after thrombectomy. |
| **Methods** | 6(b) | Cohort study - For matched studies, give matching criteria and number of exposed and unexposed. | N/A | Not applicable because this was not a matched cohort study. |
| **Methods** | 7 | Clearly define all outcomes, exposures, predictors, potential confounders, and effect modifiers. Give diagnostic criteria, if applicable. | pp. 3-5 | CTP mismatch volume and imaging definitions are provided in Section 2.2; END is defined in Section 2.4; clinical, laboratory, imaging, and treatment-related variables are listed in Section 2.5; analytic criteria are described in Section 2.6. |
| **Methods** | 8 | For each variable of interest, give sources of data and details of methods of assessment or measurement. Describe comparability of assessment methods if there is more than one group. | pp. 3-4 | Sections 2.2-2.5 describe CTP acquisition and RAPID processing, thrombectomy assessment, NIHSS assessment, electronic medical record data collection, and ECASS-based hemorrhagic transformation classification. |
| **Methods** | 9 | Describe any efforts to address potential sources of bias. | pp. 2-5 | Potential bias was addressed through consecutive enrollment, predefined eligibility criteria, standardized CTP processing and NIHSS assessments, independent review of hemorrhagic transformation, time-specific predictor eligibility, multiple imputation for missing data, and bootstrap internal validation. |
| **Methods** | 10 | Explain how the study size was arrived at. | pp. 2-3 | The study included all consecutively eligible patients treated during the study period. Of 612 screened patients, 438 met the eligibility criteria and entered the primary analysis (Fig. 1). |
| **Methods** | 11 | Explain how quantitative variables were handled in the analyses. If applicable, describe which groupings were chosen and why. | pp. 4-5 and 7-9 | Section 2.6 describes presentation and tests for normally and non-normally distributed variables; Section 3.5 explains RCS-guided mismatch volume categories (<75 mL, 75-105 mL, >105 mL); Table 5 reports grouped and continuous predictors. |
| **Methods** | 12(a) | Describe all statistical methods, including those used to control for confounding. | pp. 4-5 and 8-10 | Section 2.6 describes descriptive and group-comparison tests, multiple imputation, RCS analysis, prespecified covariate selection, multivariable logistic regression, VIF assessment, ROC analysis, calibration, DCA, bootstrap internal validation, and LASSO sensitivity analysis. |
| **Methods** | 12(b) | Describe any methods used to examine subgroups and interactions. | N/A | Not applicable. The manuscript does not report formal subgroup analyses or interaction tests. |
| **Methods** | 12(c) | Explain how missing data were addressed. | pp. 4-5 | Section 2.6 states that key variables were complete and missingness for each other covariate was below 10%. Missing values were handled by multiple imputation by chained equations (m = 20), with estimates combined using Rubin's rules. Supplementary Table 1 reports missing-data patterns, and complete-case and worst-case analyses are reported as sensitivity analyses. |
| **Methods** | 12(d) | Cohort study - If applicable, explain how loss to follow-up was addressed. | pp. 2-4 | The outcome window was limited to 72 h. Patients for whom both 72-h NIHSS and survival status could not be ascertained were excluded; deaths within 72 h were retained and classified as END events. No longer-term follow-up was analyzed. |
| **Methods** | 12(e) | Describe any sensitivity analyses. | pp. 4-5 and 8-10 | Section 2.6 prespecifies complete-case and worst-case sensitivity analyses and LASSO logistic regression. Section 3.6 and Figs. 6-7 report analyses counting early deaths as END, excluding deaths within 72 h, complete-case analysis, worst-case classification of indeterminate outcomes, and LASSO results. |
| **Results** | 13(a) | Report numbers of individuals at each stage of study, for example numbers potentially eligible, examined for eligibility, confirmed eligible, included in the study, completing follow-up, and analyzed. | p. 2 | Fig. 1 reports 612 patients screened, 174 excluded with reasons, and 438 included in the primary analysis. For the 24-h landmark analysis, 51 patients with END at or before 24 h were excluded; 387 patients entered the reassessment cohort, and 36 developed delayed END after >24-72 h. |
| **Results** | 13(b) | Give reasons for non-participation at each stage. | p. 2 | Fig. 1 reports each exclusion reason and count: posterior-circulation occlusion (n = 68), absent or unanalyzable preprocedural CTP (n = 39), prestroke mRS >2 (n = 22), severe comorbidity or terminal malignancy (n = 15), other severe intracranial lesions (n = 10), onset-to-puncture time >24 h (n = 7), and inability to ascertain both 72-h NIHSS and survival status (n = 13). |
| **Results** | 13(c) | Consider use of a flow diagram. | p. 2 | Participant selection and flow are presented in Fig. 1 (patient selection flowchart), including the numbers screened, excluded with reasons, included in the primary analysis, and included in the 24-h landmark cohort. |
| **Results** | 14(a) | Give characteristics of study participants and information on exposures and potential confounders. | pp. 5-7 | Tables 1-4 report demographic, clinical, laboratory, imaging, and treatment-related characteristics by END status. |
| **Results** | 14(b) | Indicate number of participants with missing data for each variable of interest. | pp. 4-5 and Supplementary Table 1 | Section 2.6 states that key variables were complete and missingness for each other covariate was below 10%; Supplementary Table 1 describes the missing-data patterns. |
| **Results** | 14(c) | Cohort study - Summarise follow-up time, for example average and total amount. | p. 4 | END was assessed within 72 hours after mechanical thrombectomy, with scheduled NIHSS evaluations at 2, 24, 48, and 72 hours after surgery. |
| **Results** | 15 | Cohort study - Report numbers of outcome events or summary measures over time. | pp. 5 and 7-9 | Section 3.1 reports 87 END events among 438 patients (19.9%), including 78 patients with neurological worsening and 9 deaths within 72 h. Section 3.5 reports END frequencies across mismatch-volume categories, and Section 3.7 reports delayed END after >24-72 h across the 24-h reassessment risk categories. |
| **Results** | 16(a) | Give unadjusted estimates and, if applicable, confounder-adjusted estimates and their precision. Make clear which confounders were adjusted for and why they were included. | pp. 5-6 and 8-9 | Tables 1-4 report unadjusted group comparisons. Section 2.6 explains that candidate covariates were specified in advance from prior evidence, clinical importance, and availability at the prediction time. Table 5 reports crude, selected-covariate-adjusted, and fully adjusted ORs with 95% CIs and identifies the covariates included in Models 2 and 3. |
| **Results** | 16(b) | Report category boundaries when continuous variables were categorized. | pp. 3 and 7-9 | Mismatch volume was categorized exploratorily as <75 mL, 75-105 mL, and >105 mL based on the RCS curve and observed risk pattern; collateral score was categorized as 0-1 versus 2-3. Table 5 reports ischemic core volume per 10 mL and admission NIHSS per 1 point. |
| **Results** | 16(c) | If relevant, consider translating estimates of relative risk into absolute risk for a meaningful time period. | pp. 5 and 7-9 | The manuscript reports absolute END incidence within 72 hours overall and by mismatch-volume subgroup. |
| **Results** | 17 | Report other analyses done, for example analyses of subgroups and interactions, and sensitivity analyses. | pp. 7-10 | Other analyses include RCS and correlation analyses, ROC analysis, calibration, DCA, bootstrap internal validation, sensitivity analyses addressing deaths and missing data, and LASSO sensitivity analyses (Sections 3.5-3.6; Figs. 3-7; Supplementary Tables 2-4). No formal subgroup or interaction analyses were reported. |
| **Discussion** | 18 | Summarise key results with reference to study objectives. | pp. 9-10 | The Discussion summarizes the 19.9% END frequency, the nonlinear association between mismatch volume and END, the time-separated baseline and 24-h models, and the association of parenchymal hematoma with delayed END. |
| **Discussion** | 19 | Discuss limitations of the study, taking into account sources of potential bias or imprecision. Discuss both direction and magnitude of any potential bias. | pp. 10-11 | The limitations address selection and information bias, residual confounding and measurement error, internal derivation of categories and scores, restricted applicability of the 24-h landmark model, confounding by indication, the missing-at-random assumption, imprecision from few outcome events, and limited transportability across CTP platforms and processing settings. |
| **Discussion** | 20 | Give a cautious overall interpretation of results considering objectives, limitations, multiplicity of analyses, results from similar studies, and other relevant evidence. | pp. 9-11 | The Discussion interprets the findings in relation to mismatch volume, core infarct volume, collateral circulation, recanalization, hemorrhagic transformation, model performance, and limitations. |
| **Discussion** | 21 | Discuss the generalisability or external validity of the study results. | p. 11 | The manuscript states that the proposed thresholds, scores, and potential applications require validation in independent multicenter cohorts and notes that CTP platform and processing differences may limit transportability. |
| **Other information** | 22 | Give the source of funding and the role of the funders for the present study and, if applicable, for the original study on which the present article is based. | p. 12 | The Funding section states that the research received no external funding. |

Note: Page numbers refer to the visible page numbers in the latest manuscript file (13 pages). "N/A" indicates an item not applicable to this study design.
