# Supplementary material for: Risk Stratification for Early Neurological Deterioration After Mechanical Thrombectomy and Development of a 24-h Postprocedural Reassessment Model in Patients With Acute Anterior-Circulation Large-Vessel Occlusion Based on Computed Tomography Perfusion Mismatch Volume
Source: Rev Neurol. 2026 Aug 26;81(8):53745. doi: 10.31083/RN53745 (PMC13541423; doi:10.31083/RN53745)
Supplement: Supplementary file 1 [file 1576-6578-81-8-53745-s1.zip › Supplementary Material/Supplementary Tables.docx]

**Supplementary Tables**

**Supplementary Table 1. Missing-Data Report and Distribution by Group**

| **Variable** | **Total missing, *n* (%)** | **Missing in END group, *n* (%)** | **Missing in non-END group, *n* (%)** | **Between-group *P* value** | **Explanation** |
| --- | --- | --- | --- | --- | --- |
| Admission NIHSS; CTP core, hypoperfusion, and mismatch volumes; mTICI; END | 0 (0.0) | 0 (0.0) | 0 (0.0) | — | Key variables complete |
| Random blood glucose at admission | 6 (1.4) | 1 (1.1) | 5 (1.4) | >0.999 | Occasional laboratory missingness |
| HbA1c | 31 (7.1) | 8 (9.2) | 23 (6.6) | 0.390 | Not routinely required in emergency evaluation |
| D-dimer | 24 (5.5) | 6 (6.9) | 18 (5.1) | 0.511 | Extended coagulation test |
| Fibrinogen | 18 (4.1) | 4 (4.6) | 14 (4.0) | 0.761 | Limited missingness in coagulation testing |
| LDL-C | 14 (3.2) | 2 (2.3) | 12 (3.4) | 0.744 | Limited missingness in lipid testing |
| HDL-C | 15 (3.4) | 3 (3.4) | 12 (3.4) | 0.986 | Same missingness pattern as LDL-C |
| Little's MCAR test | — | — | — | 0.418 | MCAR not rejected; MICE *m* = 20 |

**Note:** END, early neurological deterioration; CTP, computed tomography perfusion; mTICI, modified Thrombolysis in Cerebral Infarction; NIHSS, National Institutes of Health Stroke Scale; HbA1c, glycated hemoglobin A1c; LDL-C, low-density lipoprotein cholesterol; HDL-C, high-density lipoprotein cholesterol; MCAR, missing completely at random; MICE, multiple imputation by chained equations. Data are presented as number (percentage) [*n* (%)]. Between-group *P* values compare the proportions of missing data in the END and non-END groups. Little's MCAR test yielded *P* = 0.418, indicating that the MCAR hypothesis was not rejected; this does not prove that the data were missing completely at random.

**Supplementary Table 2. Sensitivity Analyses**

| **Sensitivity analysis** | ***n*** | **No. of END events** | **Primary effect** | **Apparent AUC** | **Bootstrap-corrected AUC** |
| --- | --- | --- | --- | --- | --- |
| Primary analysis: early deaths classified as END | 438 | 87 | Mismatch volume <75 mL: OR = 3.08 (1.59–5.96), *P* = 0.001 | 0.789 | 0.774 |
| Excluding deaths within 72 h | 429 | 78 | Mismatch volume <75 mL: OR = 2.94 (1.50–5.74), *P* = 0.001 | 0.781 | 0.766 |
| Complete-case analysis | 392 | 75 | Mismatch volume <75 mL: OR = 3.15 (1.63–6.10), *P* = 0.003 | 0.786 | 0.771 |
| Worst-case analysis: indeterminate outcomes classified as END | 451 | 100 | Mismatch volume <75 mL: OR = 2.88 (1.53–5.42), *P* = 0.001 | 0.776 | 0.760 |

**Note:** END, early neurological deterioration; OR, odds ratio; CI, confidence interval; ROC, receiver operating characteristic; AUC, area under the ROC curve. The primary analysis classified deaths within 72 h as END. The complete-case analysis did not use imputed data. The worst-case analysis classified patients with indeterminate outcomes as END. ORs and 95% CIs were obtained from the corresponding logistic regression models; bootstrap-corrected AUCs were based on internal resampling.

**Supplementary Table 3. Performance of the Preprocedural Baseline and 24-h Landmark Reassessment Models and Exploratory Simplified Scoring Systems**

| **Module/item** | **Specific value or points** | **Interpretation or management suggestion** | **Statistical note** |
| --- | --- | --- | --- |
| Model performance: preprocedural baseline model | *n* = 438; 87 END events during 0–72 h; AUC, 0.789 (0.735–0.843); optimal cutoff, 0.190; sensitivity, 72.4%; sensitivity, 72.4%; specificity, 73.8%; PPV, 40.8%; NPV, 91.1%; H–L P = 0.412; corrected AUC, 0.774 | Preprocedural risk stratification | Preprocedural variables only |
| Model performance: 24-h reassessment model | *n* = 387; 36 delayed END events during >24–72 h; AUC, 0.838 (0.773–0.903); optimal cutoff, 0.112; sensitivity, 77.8%; specificity, 79.5%; PPV, 28.6%; NPV, 97.1%; H–L P = 0.536; corrected AUC, 0.821 | 24-h risk re-stratification | For patients without END at or before 24 h |
| Core variables in the 24-h reassessment model | Unsuccessful reperfusion: OR = 2.12, *P* = 0.050; HI: OR = 1.22, *P* = 0.687; PH: OR = 4.18, *P* = 0.003 | PH indicates a higher risk of delayed END | HT subtypes are not included in the preprocedural model |
| Preprocedural points: mismatch volume | >105 mL = 0; 75–105 mL = 1; <75 mL = 3 | Converted from *β* coefficients | Exploratory stratification |
| Preprocedural points: ischemic core volume | <30 mL = 0; 30–50 mL = 1; >50 mL = 2 | Consistent with the distribution of core volume | Exploratory stratification |
| Preprocedural points: admission NIHSS score | ≤10 = 0; 11–16 = 1; 17–22 = 2; ≥23 = 3 | Based on severity and quartile range | Exploratory stratification |
| Preprocedural points: collateral score | 2–3 = 0; 0–1 = 2 | *β* for poor collaterals ≈ 0.80 | Exploratory stratification |
| Preprocedural low risk | 0–3 points; *n* = 163; END, 13 (8.0%) | Standard stroke-unit pathway | *P* for trend < 0.001 |
| Preprocedural intermediate risk | 4–6 points; *n* = 179; END, 37 (20.7%) | Enhanced neurological assessment during the first 48 h | *P* for trend < 0.001 |
| Preprocedural high risk | 7–10 points; *n* = 96; END, 37 (38.5%) | Continued monitoring in a stroke unit/neurological ICU through 72 h | *P* for trend < 0.001 |
| 24-h reassessment points | Preprocedural score + 2 points for unsuccessful reperfusion + 1 point for HI + 4 points for PH; total, 0–15 points | Re-stratification after 24 h | Exploratory stratification |
| 24-h low risk | 0–4 points; *n* = 221; delayed END, 8 (3.6%) | Routine monitoring through 72 h | *P* for trend < 0.001 |
| 24-h intermediate risk | 5–8 points; *n* = 113; delayed END, 14 (12.4%) | Enhanced neurological assessment and control of blood pressure fluctuations | *P* for trend < 0.001 |
| 24-h high risk | ≥9 points; *n* = 53; delayed END, 14 (26.4%) | Prompt repeat imaging according to clinical changes | *P* for trend < 0.001 |

**Note:** END, early neurological deterioration; ROC, receiver operating characteristic; AUC, area under the ROC curve; CI, confidence interval; PPV, positive predictive value; NPV, negative predictive value; H–L, Hosmer–Lemeshow; OR, odds ratio; HI, hemorrhagic infarction; PH, parenchymal hematoma; ICU, intensive care unit; *β*, regression coefficient. Sensitivity and specificity were calculated at the cutoff corresponding to the Youden index. Bootstrap-corrected AUCs were based on 1,000 internal resamples. The risk strata and monitoring suggestions in this table are exploratory descriptions from this cohort and do not represent treatment-decision thresholds.

**Supplementary Table 4. LASSO Sensitivity Analyses**

| **Model** | **Selected variables** | **AUC (95% CI)** | **Bootstrap optimism** |
| --- | --- | --- | --- |
| Preprocedural *λmin* | Mismatch volume, ischemic core volume, admission NIHSS score, collateral score, age, and random blood glucose at admission | 0.798 (0.745–0.851) | 0.029 |
| Preprocedural *λ1se* | Mismatch volume, ischemic core volume, admission NIHSS score, and collateral score | 0.786 (0.733–0.839) | 0.025 |
| 24-h *λmin* | Preprocedural score, successful reperfusion, PH, and maximum SBP >180 mmHg during the first 24 h after the procedure | 0.842 (0.776–0.908) | 0.033 |
| 24-h *λ1se* | Preprocedural score, successful reperfusion, and PH | 0.831 (0.765–0.897) | 0.028 |

**Note:** ROC, receiver operating characteristic; AUC, area under the ROC curve; CI, confidence interval; LASSO, least absolute shrinkage and selection operator; *λmin*, penalty parameter associated with the minimum error in 10-fold cross-validation; *λ1se*, more parsimonious penalty parameter selected using the 1-standard-error rule; NIHSS, National Institutes of Health Stroke Scale; PH, parenchymal hematoma; SBP, systolic blood pressure. Bootstrap optimism is the estimated degree to which apparent performance was overestimated on internal resampling.
